# Designing Deep Learning Hardware Accelerator and Efficiency Evaluation

**DOI:** 10.1155/2022/1291103

**Published:** 2022-07-13

**Authors:** Zhi Qi, Weijian Chen, Rizwan Ali Naqvi, Kamran Siddique

**Affiliations:** ^1^Department of Information and Communication Technology, School of Computing and Data Science, Xiamen University Malaysia, Sepang 43900, Malaysia; ^2^Department of Intelligent Mechatronics Engineering, Sejong University, Seoul 05006, Republic of Korea

## Abstract

With the swift development of deep learning applications, the convolutional neural network (CNN) has brought a tremendous challenge to traditional processors to fulfil computing requirements. It is urgent to embrace new strategies to improve efficiency and diminish energy consumption. Currently, diverse accelerator strategies for CNN computation based on the field-programmable gate array (FPGA) platform have been gradually explored because they have edges of high parallelism, low power consumption, and better programmability. This paper first illustrates state-of-the-art FPGA-based accelerator design by emphasizing the contributions and limitations of existing research works. Subsequently, we demonstrated significant concepts of parallel computing (PC) in the convolution algorithm and discussed how to accomplish parallelism based on the FPGA hardware structure. Eventually, with the proposed CPU+ FPGA framework, we performed experiments and compared the performance against traditional computation strategies in terms of the operation efficiency and energy consumption ratio. The results revealed that the efficiency of the FPGA platform is much higher than that of the central processing unit and graphics processing unit.

## 1. Introduction

With the striking expansion of the Internet and the swift development in the big data era, artificial intelligence has been widely developed and used in the past thirty years [1]. People's continuous significant breakthroughs in deep learning algorithms have brought the rapid evolution of artificial intelligence technology which play an essential role in the considerable variety of machine learning algorithms. Deep learning is a technology that simulates human brain neural transmission, extracts feature hierarchically, and continuously adjusts parameters from experience and data. It has a strong learning ability. The training of deep learning network model parameters needs the support of massive data and supercomputing power. We must find a better hardware computing acceleration scheme to meet the increasing amount of data and the expanding network scale. For deep learning, the central processing unit (CPU) is highly flexible, but the computing power is insufficient, and for the accelerator, the situation is entirely opposite. A combination of CPU and accelerator is a popular solution to balance flexibility and efficiency [2]. Meanwhile, manufacturers are studying the architecture of various deep learning accelerators, including GPU accelerators and DSP accelerators.

At present, the most widely used solution is to use a graphics processing unit (GPU) cluster as a heterogeneous computing system composed of general computing graphics processing unit (GPUPU) and CPU for computing acceleration. However, there are some problems in using GPU clusters. With the continuous expansion of cluster scale, colossal power consumption and heat dissipation have become the limiting factors of its scale expansion. Therefore, we need to find a better acceleration scheme with higher computing performance and lower energy consumption. A field-programmable gate array (FPGA) fully meets our high performance and low power consumption requirements as a new heterogeneous parallel computing acceleration device [3]. As a newly developing hardware architecture, FPGA has its unique advantages. With the continuous improvement of FPGA, it has an excellent performance power consumption ratio compared with GPU.

In addition, FPGA developers can flexibly program hardware according to specific applications, and by comparison, the structure of GPU cannot be altered once the design is completed. Lack of flexibility makes GPUs unable to apply well to most scenarios. Simultaneously, the emergence of advanced synthesis tools, such as high-level synthesis (HLS) and OpenCL, further shortens the cycle of developing a convolutional neural network (CNN) accelerator on FPGA. In recent years, FPGA has been deployed on a large scale in the data centers of Microsoft, Amazon, Alibaba, and other companies. Nowadays, cloud computing servers only not provide deep learning computation acceleration with conventional GPU but also supply customizable FPGA plans. In addition, researchers also start to apply FPGA in the domain of edge computing such as auto pilot and the industrial robot [4]. Although the accelerator based on FPGA has attracted attention in deep learning, nonignorable weaknesses still exist to overcome. Hence, the design of the FPGA deep learning accelerator needs to be further improved.

At present, most researches accelerate the hardware based on the forward propagation characteristics of neural network. By analyzing the parallelism of convolution algorithm of neural network, we can adapt the flexible programming of FPGA to reconstruct the suitable framework in order to achieve optimal utilization of the parallelism of convolutional algorithm. This approach can essentially help to further improve the computing speed and efficiency.

## 2. Related Work

Hardware acceleration is to make full use of the inherent characteristics of hardware, use hardware computing module to replace the software algorithm run by general CPU, share the workload of CPU, and improve the overall efficiency. Typical hardware accelerations include ASIC, FPGA, and GPU. Because FPGA not only has the advantages of high speed and stability like ASIC but also has the flexibility of customizing circuit structures like a software program, FPGA has attracted the attention of most scholars [4].

Chen et al. [5] proposed that matching computing throughput and memory bandwidth should be fully considered in implementing the CNN model on FPGA. Therefore, they designed the analysis method according to the roof model. The experiment implemented a CNN model on the VC707 FPGA development board. The results showed that the peak performance was 61.62 GOP/s at 100 MHz, which was better than the previous work.

Li et al. [6] proposed a way to find parallel strategies for each layer. In addition, with two distinctive matrix calculation modes on the FC layer, this design can significantly reduce the on-chip cache space required. Finally, the AlexNet network was run on Xilinx VC709, which realized the peak performance of 565.94 GOP/s and 391 fps at 156 MHz clock frequency.

Kalms et al. [7] conducted a comparative study on implementing feature detection algorithm using OpenCL on a heterogeneous system including CPU, GPU, and FPGA, and compared differences in energy efficiency and architecture performance under different optimization strategies. It showed how to use Xilinx's Edexcel tool to maximize performance and maintain low resource utilization on the premise that FPGA reached the bandwidth limit. The results demonstrated the speed of FPGA was increased by 62.8 times, and the energy consumption was reduced by 49 times in comparison with the optimized single threaded CPU.

Liang et al. [8] pointed out that the traditional CNN algorithm (i.e., spatial convolution algorithm and GEMM algorithm) is often limited by the number of computing units of FPGA. Winograd fast algorithm can still reduce the computational complexity significantly. The authors first proposed a new method of deploying the Winograd algorithm on FPGA to reuse data using line buffer structure efficiently. At the same time, they also adopted pipeline and parallel technology. They proposed an analysis model for complex design space to predict resource utilization and performance. The experiments obtained the convolutional layer average performance of 1006.4 GOP/s and the average performance of 854.6 GOP/s on AlexNet using Xilinx ZCU102 development board. The convolutional layer average performance of 3044.7 GOP/s and the overall average performance of 2940.7 GOP/s were obtained on VCC 16.

Cong et al. [9] of the University of California analyzed the characteristics of convolution operation from the mathematical level and used matrix multiplication to calculate convolution. In their study, several optimization methods for convolution matrix multiplication were proposed and compared with traditional matrix multiplication.

Intel proposed the FPGA deep learning accelerator (DLA) based on Intel OpenCL [10]. The accelerator adopted the Winograd algorithm and used the feature that a DSP on the Altera platform was able to handle the multiplication of two half-precision floating-point numbers. DLA adopted a one-dimensional Winograd algorithm in the specific implementation which reduced the multiplication times significantly. Finally, the performance of 1.38 TOP/s was achieved on the Intel Altera Aria 10 platform.

## 3. Field-Programmable Gate Array Architecture

**Figure 1:**
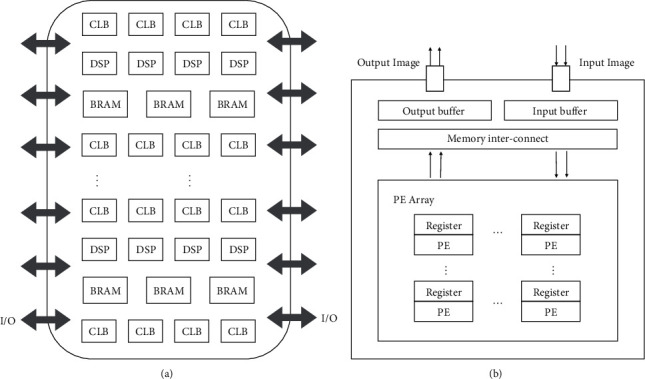
(a) FPGA chips' architecture [3]; (b) the processing flow running on FPGA architecture.

A field-programmable gate array (FPGA) is a kind of integrated circuit, which can be modified repeatedly to design different circuit functions [3]. Designers can achieve the desired logic operation by configuring the switch state. Symbolically, FPGA acts as a pile of building blocks. Developers can reconstruct its hardware structure through programming to build FPGA into any shape required. Programmable logic (PL), the FPGA's core part, is mainly comprised of the configurable logic block (CLB) and input/output block (IOB). As shown in Figure 1(a), CLBs are arranged in a two-dimensional array. Each CLB includes two logic slices and is adjacent to a switch matrix. Logic slice is mainly composed of the lookup table and flip-flop. They can be configured into different composition logic, which gives FPGA reconfigurable characteristics.

Figure 1(b) shows the basic design of the CNN FPGA accelerator. From the perspective of data, FPGA can be roughly divided into three layers: off-chip storage, on-chip cache, and register [11]. The off-chip storage usually uses DRAM memory, and the on-chip cache is block RAM. Because the on-chip cache cannot store the whole input dataset, it usually holds part of the data. The input buffer relates to the computing unit through the data transmission control unit, which transmits data required to the internal register of the computing unit each time. FPGA has the characteristics of parallel computing. Many internal computing units can calculate simultaneously, and the efficiency can be significantly improved.

FPGA is widely used in more and more fields because of its high programming flexibility and powerful computing performance. Compared with GPU, FPGA has the following advantages:

1. FPGA uses fine-grained parallel architecture. After determining the computing clock cycle time method, the designer can establish a more optimized output mode that eliminates the need to save data in the main memory to reduce memory reading delay.
2. Compared with GPU, FPGA has muscular programming flexibility. At the same time, FPGA supports dynamic reconfiguration of the algorithm, which has strong reconfigurability.
3. FPGA is much lower than GPU in power consumption and has better performance under the same power consumption, which can significantly reduce the heat dissipation problem of the processor.

## 4. Convolutional Neural Network

The CNN model imitates the structure of a biological vision system and is a feedforward artificial neural network. The core idea of CNN has three points: local perception, weight sharing, and pooling [13].

Local perception refers to the connectivity of network parts. Every neuron is only connected with part of the upper layer neurons and senses the local part instead of the entire image. The intermediary of upper and lower neurons is called the “convolutional kernel.” Weight sharing implies that all neurons use the same weight in the same convolutional kernel, remarkably reducing the number of network parameters and making it feasible to train large-scale networks. The model can learn from a local region and then convolute the whole image with the same convolution kernel. Pooling is a kind of downsampling that will separate the input images into a few rectangular regions and outputs the maximum values for each sub-region.

CNN comprises three common layers: convolutional layer, pooling layer, and fully connected layer. In general, the convolutional layer is the principal layer in the CNN model. It performs convolution operations, like the image filtering function. Convolution is a local operation that “rolls” on the feature map through a specific convolution kernel size to realize the feature extraction of the image. The pooling layer is a process of dimension reduction and extraction of the visual input image based on a biological vision system. The fully connected layer classifies the data output in a CNN model.

### 4.1. CNN Multilayer Computing Model

The CNN model selected in this paper is AlexNet model, which is the champion of ImageNet competition in 2012 [14]. It contains five convolutional layers, three fully connected layers, and three maximum pooling layers. Each layer of the network uses the ReLU activation function except the last one.  Table 1 shows the parameters of AlexNet model structure. *N*_in_ is the number of input feature maps, *N*_out_ is the number of output feature maps, Size_in_ and Size_out_ represent the sizes of input feature maps and output feature maps, Size_kernel_ is convolution kernel's size, and Stride is window sliding size in the process of convolution and pooling.

Convolution is the most important part of CNN model and the most computationally intensive part, which usually accounts for more than 90% of the computation of the whole neural network. Convolution means the process of accumulating by the product of input feature maps *N*_*if*_ and *K* × *K* convolutional kernels, and corresponding eigenvalues are extracted and output as neuron parameters, shown in (1). out(*f*_*o*_, *x*, *y*) is the value at (*x*, *y*) in the output feature map *f*_*o*_, *wt*(*f*_*o*_, *f*_*i*_, *f*_*x*_, *f*_*y*_) represents the weight of the corresponding position in the convolutional kernel, in(*f*_*i*_, *x*, *y*) is the value at (*x*, *y*) in the input feature map *f*_*i*_, while *k*_*x*_ is the *x* value of the *kth* kernel and *k*_*y*_ is the *y* value of the *kth* kernel.

(1) $$\begin{array}{c} \text{out}\left(f_{o},x,y\right)=\sum_{f_{i}=0}^{N_{if}}\sum_{k_{x}=0}^{K}\sum_{k_{y}}^{K}wt\left(f_{o},f_{i},f_{x},f_{y}\right)\times \text{in}\left(f_{i},x+k_{x},y+k_{y}\right). \end{array}$$

Pooling is aimed to lessen the size of network feature maps and decrease the computational complexity and the number of parameters while retaining the main features. The commonly used pooling method in CNN is maximum pooling. As shown in (2), this process calculates the maximum value of *K* × *K* adjacent neurons in the feature map.

(2) $$\begin{array}{c} \text{out}\left(f_{o},x,y\right)=\text{max}\left(\text{in}\left(f_{o},x+k_{x},y+k_{y}\right)\right). \end{array}$$

The fully connected layer is where to make classification, each of input neurons and output neurons is fully connected, and the values of output neurons are weighted sums of all input neurons, as shown in

(3) $$\begin{array}{c} \text{out}\left(f_{o}\right)=\sum_{f_{i}=0}^{N_{if}}wt\left(f_{o},f_{i}\right)\times \text{in}\left(f_{i}\right). \end{array}$$

### 4.2. CNN Computational Complexity Analysis

In the forward propagation calculation of single-layer network in CNN, we can estimate its time complexity by multiplication operations times [13]. In a convolutional layer, each convolutional kernel is a *k* × *k* filter, which is applied to feature maps with the size of *w* × *h*. The number of convolutional kernels is *N*_in_ × *N*_out_; therefore, the time complexity of a convolution layer can be expressed as

(4) $$\begin{array}{c} C_{\text{CONV}}^{\text{Time}}=O\left(N_{\text{in}}\times N_{\text{out}}\times k^{2}\times w\times h\right). \end{array}$$

For the pooling layer, the number of input feature maps *N*_in_ and number of output feature maps *N*_out_ are equal, so the time complexity of the pooling layer and the fully connected layer can be

(5) $$\begin{array}{c} C_{\text{Pooling}}^{\text{Time}}=O\left(N_{\text{in}}\times w\times h\right),\\ C_{FC}^{\text{Time}}=O\left(N_{\text{in}}\times N_{\text{out}}\right). \end{array}$$

From these equations, it can be clearly seen that the calculation of the convolutional layer in the CNN model occupies the primary position. Its time complexity is significantly higher than that of a fully connected layer. Therefore, we need to focus on how to accelerate the calculation of the convolution layer. Due to the unique structure of FPGA, the parallelism of computing can be realized through programming. We can consider realizing CNN parallel computing to reduce the time complexity and achieve the purpose of acceleration.

### 4.3. CNN Computing Parallelism Analysis

The core task of heterogeneous computing acceleration based on FPGA is to extract the parallelism characteristics of the network in the computing process. The CNN model consists of multiple layers. Hence, our first thought is to realize the parallelism of various layers [15]. However, in the calculation process of CNN, due to the transmission characteristics of data forward propagation, the data between adjacent layers have a strong correlation, which dramatically limits the computational parallelism between layers, making it difficult to achieve task parallelism between layers. Despite the low parallelism between different layers, there is high parallelism in the single-layer calculation. Moreover, each layer of CNN has a similar network structure, and the computation of each layer has a high degree of similarity. Therefore, the whole convolutional neural network can be achieved by completing the single-layer network design and then reusing these computation resources.

**Figure 2:**
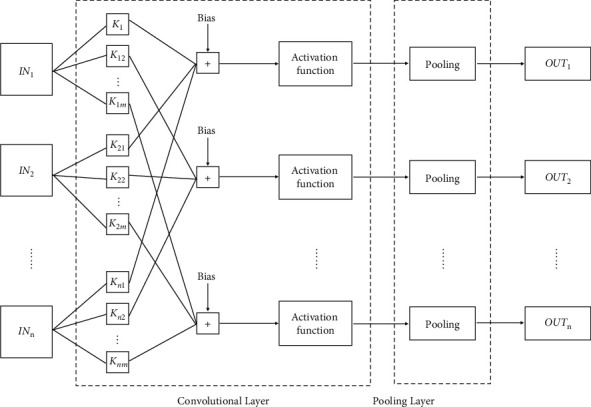
A schematic diagram of single-layer network computing process.

The procedure of CNN single-layer operations is shown in Figure 2, where IN_1_, IN_2_,…, IN_*n*_ are the *n* input feature maps in this layer, OUT_1_, OUT_2_,…, OUT_*n*_ are *m* output feature maps in this layer, and *K*_11_ − *K*_*nm*_ are *n* × *m* convolution kernels. Different layers in CNN have different values for *n* and *m*, and each feature map is associated with the corresponding convolutional kernel *K*_*nm*_, performs convolution, accumulates the results, and adds the offset value, and then obtains the output of the convolutional layer through the nonlinear activation function sigmoid. The output will be used as the input of the pooling layer. After pooling, the output feature map of the layer will be obtained. In this process, CNN's single-layer computing process has high parallelism, which can be divided into the following [16].

#### 4.3.1. Parallelism in the Convolutional Kernel

**Figure 3:**
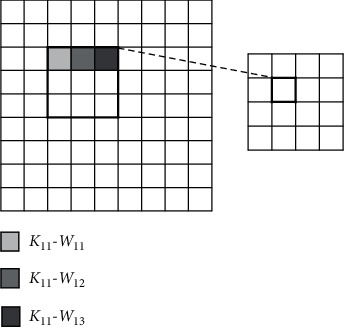
Parallelism in the convolution kernel.

When a single convolutional kernel convolutes the input feature map, due to the independent product of each pixel, the *K* × *K* th product and accumulation operation in a single convolution window can be carried out in parallel, as shown in Figure 3. The parameters *K*_*nm*_ − *W*_*xy*_ represent the weight at position (*x*, *y*) in the *n* × *m* convolutional kernels.

#### 4.3.2. Parallelism between Convolutional Kernels

**Figure 4:**
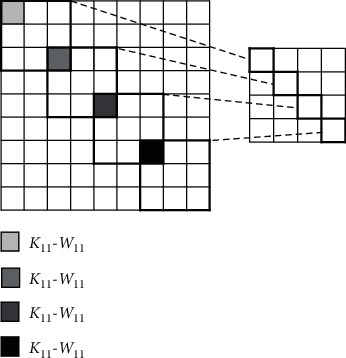
Parallelism between convolution kernels.

For multiple pixels on the same input feature map, the identical convolutional kernels can execute in parallel, and multiple neurons of the output feature maps can be obtained at the same time, as shown in Figure 4.

#### 4.3.3. Inter-Layer Parallelism

**Figure 5:**
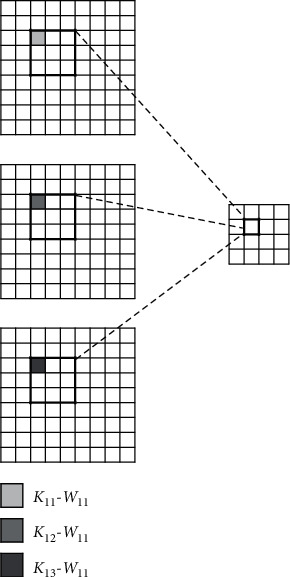
Inter-layer parallelism.

Figure 5 shows that each neuron in output feature maps corresponds to the product accumulation result of *n* input feature maps and various convolutional kernels. For the exact position in these distinct input feature maps, the convolutional kernels are able to convolute in parallel.

#### 4.3.4. Inter-Output Parallelism

**Figure 6:**
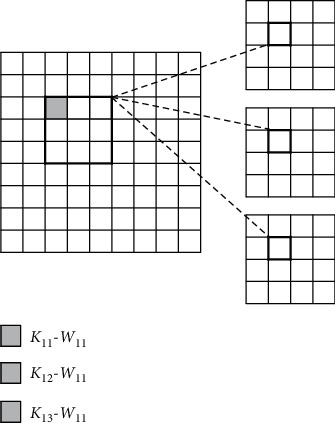
Inter-output parallelism.

The same convolutional window on the input feature map and different convolutional kernels can perform convolution operations in parallel to obtain the values of neurons at the same position of distinct output feature maps, as shown in Figure 6.

By combining the above four parallel characteristics of convolution computing, we can exploit the programmability of FPGA hardware to realize the ability of parallel convolution computing and achieve a better acceleration effect.

## 5. FPGA Parallelism Design

### 5.1. Parallel Design and Analysis of FPGA Computing Unit

There are multi-level nested loops in the convolutional module of CNN, and hundreds of convolution operations need to be carried out. If the design of parallel operation is not adopted, its time complexity will be inestimable. Generally, FPGA acceleration will consider how to optimize the loop structure. When each loop is relatively independent, the loop can be expanded to improve the degree of parallelism. Because there are many product and accumulation operations in the convolutional layer, the higher the degree of parallelism we achieve, the fewer the clock cycles, and the convolutional layer needs to complete a convolution.

However, in the design, choosing different levels of loop expansion may produce different results. Further, to what extent the expanded execution units share data which affects the complexity of the generated hardware and finally impacts the number of replicas and hardware operation frequency. Firstly, we need to use the parallelism between CNN outputs to divide the convolution calculation of multiple nested loops and apply the method of loop tiling and loop expansion to construct the calculation circuit directly used in the CNN layer. Loop tiling technology transforms the program with nested loops, reconstructs the code and tiles, and reorders the iteration space to improve the data locality and block parallelism [17]. The loop expansion technology makes use of the parallelism between convolutional kernels to carry out multiple convolutional parallel processing for the multi-input and one-output feature extraction process of the loop in the tile. This method is conducive to reducing the execution time of extracting a feature map.

### 5.2. FPGA Overall Framework Design

**Figure 7:**
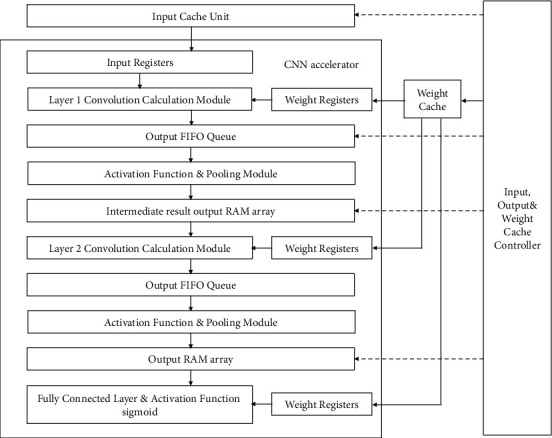
The proposed framework of FPGA hardware architecture as a CNN accelerator.

Based on the previous work [6–10], the general framework of parallel computing of convolutional neural network based on FPGA can be summarized as follows: considering forward propagation, the design division in the overall architecture is determined by using the parallel computing characteristics of convolutional neural network and loop transformation. The system framework is divided into different hierarchical structures according to the characteristics of parallel and serial processing [18].  Figure 7 shows the hardware architecture of the CNN. The subsystem of the overall architecture mainly includes input cache module, weight data storage module, convolution module, pooling module, fully connected layer, activation function module, and control module.

The control module controls the transmission sequence of data streams and communication between modules. Firstly, the control logic generates a data request signal. Under the control of the signal, the input data stream is synchronized with the system clock and updated after each cycle. Then, the input data are sent to the functional modules of each parallel or serial subsystem to complete the operation of several feature maps. Finally, after consequential feature extraction in each layer, feature classification combines multiple local feature data streams into global features and sends them to the fully connected layer module for classification.

## 6. Framework Verification and Result Analysis

The CNN heterogeneous accelerator based on FPGA discussed in this paper was implemented by OpenCL heterogeneous computing standards and aimed to complete the hardware acceleration of CNN forward propagation computing. This section tests and verifies the accelerator by using the parameters of the trained AlexNet model and images from the ImageNet dataset and then analyzes the experimental results and the performance of the FPGA accelerator system.

**Figure 8:**
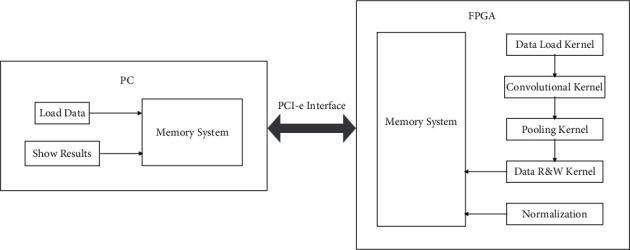
The proposed verification framework comprising PC and FPGA.

The accelerator function verification mode designed in this paper is CPU+ FPGA. The required functions are designed on FPGA and arranged into an accelerator structure in line with CNN operation. The operating system software of the computer sends the parameters required by the model and the image data to be processed to the FPGA accelerator via the PCIe bus. Afterward, the accelerator uses the PCIe bus again to send its running results back to the computer. The corresponding system will display running results and classification outcomes, and compare them with the benchmark solution to evaluate the performance of the accelerator.  Figure 8 shows the overall structure of the FPGA accelerator.

The detailed steps are as below: firstly, the input data (mainly images) will be stored on the PC, and the reverse training of the neural network model has been completed on the computer. Then, the input data and model parameters will be directly passed to the FPGA with the PCIe bus.

After that, the controller of FPGA will carry out the mission to analyze the input data and AlexNet model parameter input by PC and keep them in external memory (LDDR3). When the control layer receives control instructions from the PC, the corresponding modules of the various kernels and output layers are executed to complete the computations of the neural network. The output result is returned to the PC when the forward propagation calculation is completed.

### 6.1. Methodology

We selected the FPGA hardware, the Xilinx Kintex-7 development board XC7K325T as our experimental platform, and OpenCL as the development environment. The experimental environment and the configuration information of the devices we used are listed in Table 2.

#### 6.1.1. Model Parameter Acquisition

Here, we used the trained model CaffeNet under the Caffe framework, which is the AlexNet model [25, 26]. The design of the whole system was based on this model structure. The dataset used for model training is ImageNet [27]. Once the Caffe deep learning framework is installed, MATLAB has been used to extract the parameters of the network model.

#### 6.1.2. Input Image Acquisition and Preprocess

The images used in the test are from the ImageNet image dataset. This image dataset and corresponding category tag file synset_words.txt can be downloaded from the relevant website. The image needed to be preprocessed before testing, including image size adjustment and demean processing. The required image size was 227 × 227, and these preprocessing operations were processed by the script provided by the Caffe framework. The test method of the FPGA accelerator is to select a certain number of pictures from the ImageNet dataset for classification and record the accuracy of program classification and execution time to verify the accuracy and performance of the accelerator.

#### 6.1.3. Data Transmission Process

In PC platform, data are stored in the inner memory and transferred automatically through data buses, which means CPU and GPU do not require extra data manipulation, but the situation for FPGA is different, and the time consumption of data transmission to FPGA board memory via the PCIe bus is essential. To eliminate these cost overheads, we implemented the scheme in which data processing and transmission take place simultaneously. It is admitted that this manipulation would make an impact on the execution performance but limited to the current architecture, most of FPGA board does not have an integrated storage, and thereby, the cost of data transmitting is deemed as unavoidable. It is noteworthy that we applied PCIe bus to complete the mission, which most devices are not equipped with, and the alternative way to do is through LAN or Thunderbolt 3.

#### 6.1.4. Software Implementation

In our work, OpenCL [28] was chosen to develop the FPGA accelerator mainly because of the great convenience and efficiency it brought. The OpenCL programs could be divided into two parts, one for the PC and the other for the FPGA board. The part in the PC took responsibility for the controlling of the whole program, and the other oversaw the completion of allocated computing tasks. To achieve that, four main functions in the CNN model were rewritten as the strategy mentioned above, including memory reading/memory writing, conventional kernel, and pooling.

#### 6.1.5. Measurement Method

To compare the performance of different devices, we exploited various aspects of variables. System commands like nvidia-smi or ps were utilized to measure the behaviors of CPU and GPU. Vivado [29] was also deployed, helping monitor the FPGA board. With the hardware performance statistics, throughput was strictly calculated by the general formula. By contrast, MATLAB was employed to run the original AlexNet model as the controlled group. The accuracy of model on distinct devices was assessed automatically by the output part of the programs.

### 6.2. Result Analysis

The CNN heterogeneous accelerator structure proposed in this article achieves the image classification function, but the batch processing operation of images is not considered in the design procedure. Hence, only a single image can be classified at a time. After classifying 100 test images (randomly chosen) in the dataset, the accurate rate reached 54%, which was basically consistent with the standard results in Caffe. To evaluate the acceleration performance of the system, an experimental comparison was carried out on three platforms: FPGA, CPU, and GPU. The batch size was set to 1 to keep the conditions consistent. The test results under the three platforms were compared, as shown in the following table. The energy efficiency ratio in Table 3 refers to the ratio of speedup to power. The listed power is the rated power used for comparison purpose, and it may be different from the actual power.

In Table 3, it can be found that for the same input image data, GPU has the best performance of 4.3 s but also with the highest power consumption of 120.0 W. Compared with the CPU, the speedup of our FPGA accelerator was 8.67, and the energy efficiency was 3 times that of GPU and 157 times that of CPU. Moreover, our implementation achieved almost double throughput as much as that of the previous work [6] and even with lower energy consumption and higher energy efficiency.

Although GPU had the shortest running time and the highest speedup, the energy consumed was also the maximum. With the same power supply, FPGA tends to have a better performance than GPU. In terms of energy efficiency, the heterogeneous acceleration implementation of CNN based on the FPGA platform discussed above was better than GPU, which also implied that FPGA performed extremely well on the balance of performance and energy consumption.

## 7. Limitations and Path Forward

Although our design of deep learning accelerator is innovative and progressive, there are some limitations in our work. The section presents such limitations and puts forward possible future works.

### 7.1. Data Transmission

Up until now, unlike conventional computers, most FPGA boards do not merely integrate external storage. Hence, when it requires manipulating a vast dataset, the FPGA board cannot store all of them in advance but receives each piece of it successively, which essentially led to slow down the operation speed. Hence, in the future, with the further shift in the data transmission mode, it may produce better performance because time consumption on PCIe would likely to be demolished.

### 7.2. Performance Measurement

In the experimental evaluation, we evaluated the performance with the support of other software, and it was manifested that the software is also supposed to occupy some recourses more or less. Therefore, for the future work, it is suggested that the assessment of performance should be carried out in a more controlled and sophisticated environment such as with external gauges or appropriate virtual machines [30].

### 7.3. Experimental Implementation and Dataset Selection

To make a clear comparison between different schemes, we opted to use the same FPGA board as in the previous work [6]. Generally, replicating the hardware configurations poses difficulty as some of the resources may not be available in the market. Nevertheless, it is necessary to take the progress of FPGA architectures into consideration when measuring the speedup brought by different methods. Additionally, our work is limited to one dataset where a bias may exist. In the future work, it is suggested to utilize multiple datasets in order to further improve the comparison reliability [31].

## 8. Conclusion and Future Prospects

Several accelerator approaches for CNN computation based on the field-programmable gate array (FPGA) platform have shown great progresses in many areas because they have edges of high parallelism, low power consumption, and better programmability. In our research, we illustrated some important concepts of parallel computing (PC) in convolution algorithm and figured out how to realize parallel computing in FPGA hardware structure.

On the basis of theoretical analysis, we applied the verification model of FPGA combined with PC to verify the acceleration function of FPGA and compared the performance of FPGA with traditional computing processors in terms of operation efficiency and energy consumption ratio. The accelerator realized a complete forward propagation process of CNN, and its convolution unit combined multiple parallel computing operations to effectively improve the overall performance.

In order to verify the advantages of FPGA over traditional processors, we compared the performance against traditional computation processors related to the operation efficiency and energy consumption ratio. By combining the parallelism of CNN algorithm, FPGA significantly showed a better energy consumption ratio and efficiency than traditional CPU and GPU. For the same input image data, G GTX 1660 Ti Ultra 6G-GPU showed the best performance of 4.3 s but also with the highest power consumption of 120.0 W. Compared with the Intel i5-10400F-CPU, the speedup of our FPGA accelerator was 8.67, and the energy efficiency was 3 times that of GPU and 157 times that of CPU. At the clock frequency of 1818mhz, the peak computing rate of FPGA can reach 76.19 GOPS.

Although FPGA has made some achievements in accelerating deep learning with its advantages of reconfigurability and low energy consumption, it also has some disadvantages such as hardware programming difficulties and considerable time cost of the reconstruction process. Therefore, FPGA still has a long way to go to reach the stage of more widely used deep learning accelerators. As per the current development progress, some promising research directions can be outlined as follows:

### 8.1. Activation Function Optimization

At present, most of the research focuses on the repetition part of matrix operation in FPGA computing optimization [5, 6], but few on the improvement of activation function optimization, so this is a field to be excavated.

### 8.2. Data Optimization

The use of fixed-point data with fewer bits can improve the acceleration performance of the model, but it also brings the disadvantage of low precision [8]. Future research on dynamic precision data quantization can be strengthened to make different layers of neural networks. It requires different formats of fixed-point numbers, finding a structure of fixed-point numbers with suitable bits, and maintaining the necessary minimum accuracy.

### 8.3. Data Path Optimization

Data path optimization can be divided into on-chip and off-chip data transmission. Xuechao et al. [32] used a systolic array structure to transmit data and reduced the access of computing units to on-chip cache, making the design reach a very high frequency. Compared with today's popular accelerator GPU, the bandwidth of FPGA is limited, i.e., only 4∼20 GB/s in general. The optimization goal of most existing studies is to reduce the off-chip memory access bandwidth requirements.

### 8.4. FPGA Cluster

The performance of integrating multiple FPGA chips is very promising. However, how to deal with the processing scheduling and allocation between chips is still a significant challenge. Further research work may be initiated from various fine-grained divisions and weight allocations between multiple chips.

It can be predicted that deep learning has a broad development prospect in the future as a revolutionary implementation method of machine learning. Similarly, the deep learning acceleration technology based on FPGA is also expected to get better with each passing day and would finally promote the reform and development of the whole field of artificial intelligence.

### 8.5. New Deep Learning Model

The proposed design in this paper focused on the parallelism of CNN model. Although it was the most popular deep learning solution, the researchers are encouraged to explore other trending models too such as the transformer, proposed by Google [33]. The transformers have also shown better parallelism than CNN in many areas; therefore, the functionality of both FPGA and transformers should also be explored as it may lead to a new trend.

## Figures and Tables

**Table 1:** AlexNet model parameter architecture [14].

Layer	*N*_in_	*N*_out_	Size_in_	Size_out_	Size_kernel_	Stride
CONV1	3	96	227 × 227	55 × 55	11 × 11	4
POOL1	96	96	55 × 55	27 × 27	3 × 3	2
CONV2	48	256	27 × 27	27 × 27	5 × 5	1
POOL2	256	256	27 × 27	13 × 13	3 × 3	2
CONV3	256	384	13 × 13	13 × 13	3 × 3	1
CONV4	192	384	13 × 13	13 × 13	3 × 3	1
CONV5	192	256	13 × 13	13 × 13	3 × 3	1
POOL5	256	256	13 × 13	6 × 6	3 × 3	2
FC6	9216	4096	1 × 1	1 × 1	—	—
FC7	4096	4096	1 × 1	1 × 1	—	—
FC8	4096	1000	1 × 1	1 × 1	—	—

**Table 2:** The experimental environment.

Device	Configuration information
CPU	Intel i5-10400F [19]
Mother board	MSI Z590-A PRO 16 GB 2400 MHz [20]
Memory	HyperX Savage 8 GB DDR4 2400 [21]
GPU	iGame GeForce GTX 1660 Ti ultra 6G [22]
FPGA board	Xilinx Kintex-7 FPGA [23]
OS	Ubuntu 16.04 [24]

**Table 3:** The comparative evaluation of the existing and the proposed implementation schemes.

Experimental platform	CPU	GPU	FPGA [6]	FPGA (proposed)
Platform configuration	i5–10400F	GTX 1660Ti	V6-690T	Xilinx Kintex-7
Data type	Fp32	Fp32	Fix16	Fix16/Fp32
Clock frequency (MHz)	4300	1845	—	1818
Execution time (s)	176.2	3.9	—	20.3
Energy consumption (W)	65	120	25.6	23.3
Throughput (GOPS)	1.359	117.4	41.32	76.19
Energy efficiency (GOPS/w)	0.0209	0.978	1.65	3.27
Speedup	—	40.98	—	8.67

## Data Availability

The data used to support the findings of this study are available at https://image-net.org/download.php.
